# Mutual Exclusion of *Methanobrevibacter* Species in the Human Gut Microbiota Facilitates Directed Cultivation of a *Candidatus* Methanobrevibacter Intestini Representative

**DOI:** 10.1128/spectrum.00849-22

**Published:** 2022-06-14

**Authors:** Adrian Low, Jolie Kar Yi Lee, Jean-Sebastien Gounot, Aarthi Ravikrishnan, Yichen Ding, Woei-Yuh Saw, Linda Wei Lin Tan, Don Kyin Nwe Moong, Yik Ying Teo, Niranjan Nagarajan, Henning Seedorf

**Affiliations:** a Temasek Life Sciences Laboratory, Singapore; b Genome Institute of Singapore, A*STAR, Singapore; c Baker Heart and Diabetes Institute, Melbourne, Victoria, Australia; d Saw Swee Hock School of Public Health, National University of Singapore, Singapore; e NUS Graduate School for Integrative Science and Engineering, National University of Singapore, Singapore; f Department of Statistics and Applied Probability, National University of Singapore, Singapore; g Life Sciences Institute, National University of Singapore, Singapore; h Department of Biological Sciences, National University of Singapore, Singapore; Nanchang University

**Keywords:** Archaea, human gut microbiota, Methanobrevibacter, methanogens

## Abstract

Methanogenic Archaea (methanogens) are a phylogenetically diverse group of microorganisms and are considered to be the most abundant archaeal representatives in the human gut. However, the gut methanogen diversity of human populations in many global regions remains poorly investigated. Here, we report the abundance and diversity of gut methanogenic Archaea in a multi-ethnic cohort of healthy Singaporeans by using a concerted approach of metagenomic sequencing, 16S rRNA gene amplicon sequencing, and quantitative PCR. Our results indicate a mutual exclusion of *Methanobrevibacter* species, i.e., the highly prevalent Methanobrevibacter smithii and the less prevalent *Candidatus* Methanobrevibacter intestini in more than 80% of the samples when using an amplicon sequencing-based approach. Leveraging on this finding, we were able to select a fecal sample to isolate a representative strain, TLL-48-HuF1, for *Candidatus* Methanobrevibacter intestini. The analyzed physiological parameters of M. smithii DSM 861^T^ and strain TLL-48-HuF1 suggest high similarity of the two species. Comparative genome analysis and the mutual exclusion of the *Methanobrevibacter* species indicate potentially different niche adaptation strategies in the human host, which may support the designation of *Candidatus* M. intestini as a novel species.

**IMPORTANCE** Methanogens are important hydrogen consumers in the gut and are associated with differing host health. Here, we determine the prevalence and abundance of archaeal species in the guts of a multi-ethnic cohort of healthy Singapore residents. While Methanobrevibacter smithii is the most prevalent and abundant methanogen in the human gut of local subjects, the recently proposed *Candidatus* Methanobrevibacter intestini is the abundant methanogen in a minority of individuals that harbor them. The observed potential mutual exclusion of M. smithii and *Ca*. M. intestini provides further support to the proposal that the two physiologically similar strains may belong to different *Methanobrevibacter* species.

## OBSERVATION

Methanogenic Archaea, also called methanogens, comprise a phylogenetically diverse group of microorganisms with methanogenesis as the exclusive metabolic pathway for energy conservation (1). The presence or absence of methanogens in the human gastrointestinal tract has been associated with different health phenotypes, such as body weight variations, periodontal disease, or cardiovascular disease (2–4). However, most studies aimed at characterizing the methanogen diversity have been performed in the United States or Europe (4, 5), while the gut methanogen diversity in other regions of the world has received little attention. This presents a potential knowledge gap in the understanding of human gut methanogen diversity as different ethnicities or cultural and dietary habits may potentially affect gut microbiota composition. In this regard, Southeast Asia is of particular interest as the gut microbiota of the rapidly growing and multi-ethnic populations in this region remains vastly understudied. 

**FIG 1:**
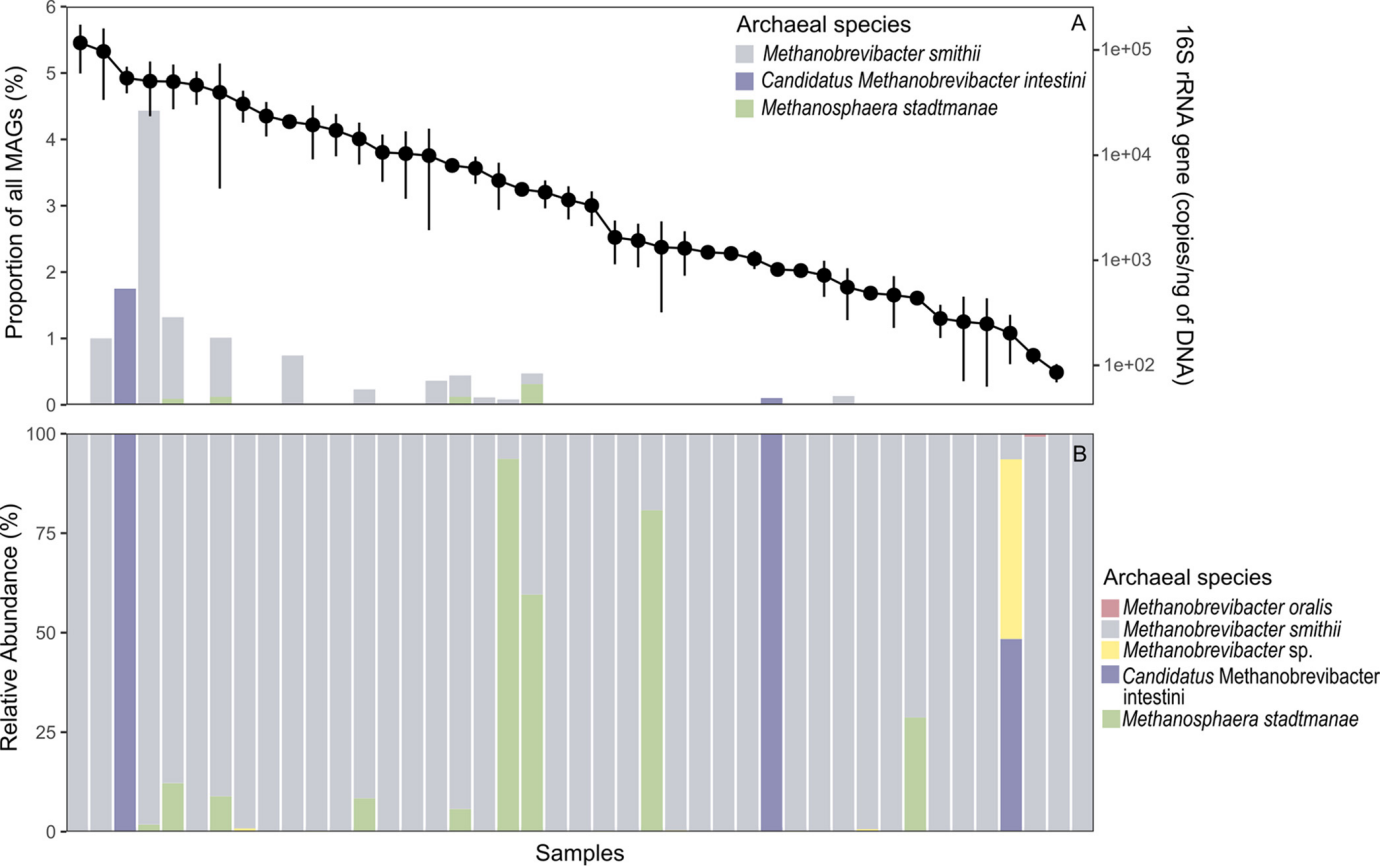
Methanogen diversity in fecal samples of multi-ethnic Singapore cohort. (A) Stacked barplot shows the relative abundance of methanogen species with MAGs (*n *= 19) of at least medium quality. The line-plot shows the abundance of archaeal 16S rRNA genes in methanogen positive samples (*n *= 43) via quantitative PCRs (qPCRs). Samples are arranged in the order of high to low abundance (left to right). One qPCR positive sample was not amplicon sequenced and was omitted from analysis. (B) Relative abundance (rarefied to 1,220 reads per sample) of methanogens in (*n *= 43) samples characterized using the same primers for qPCR via amplicon sequencing. Methanobrevibacter oralis is in a single sample at 0.8% relative abundance (amplicon sequencing).

For this study, fecal samples were collected from 109 generally healthy Singapore residents aged 48 to 76 years old (median age = 60 years old) of Chinese (*n *= 53), Indian (*n *= 30), or Malay (*n *= 26) ethnicity (see Table S1 for metadata). The relative and total abundance of methanogens as well as the taxonomic composition in samples were determined by quantitative PCR (qPCR), metagenomic sequencing (MGS), and 16S rRNA gene amplicon sequencing (AS). By using methanogen specific primers for qPCR, methanogens were detected in 44 of 109 (40.3%) samples with a maximum of 1.16 × 10^5^ ± 5.7 × 10^4^ copies/ng of DNA (data variation is expressed as standard deviation throughout the manuscript), which is within the range of positive qPCR detections in studies involving healthy individuals (5, 6) (Fig. 1A). A hybrid-assembly approach using Nanopore long- and Illumina short-sequence reads was used to construct metagenome assembled genomes (MAGs). It was possible to assemble medium-quality (contamination <10%, >50% completeness), near-complete (contamination <5%, >90% completeness) and high-quality (near-complete MAG with a defined set of rRNA and tRNA genes) MAGs in 14 of 109 samples (12.8%) (see Table S2 for MAG statistics and quality). Twelve MAGs belonging to M. smithii, two to *Candidatus* Methanobrevibacter intestini, and five to Methanosphaera stadtmanae were detected. However, the observed methanogen population as a fraction of the total gut microbiota was low with a maximum relative abundance of 4.43% M. smithii, 1.75% *Ca.* Methanobrevibacter intestini, and 0.65% *Msp. stadtmanae* (Fig. 1A). Methanogens of other orders, e.g., Methanomassilicoccales, were not obtained even when low-quality (completeness <50%) MAGs were taken into consideration. A weak correlation (rho = 0.34; Spearman’s rank, *P*-value = 0.025) between the total abundance of methanogens (qPCR) 16S rRNA genes and relative abundance of methanogen MAGs in qPCR positive samples (*n *= 44) shows that reliable MAGs are not consistently obtained from samples of high total methanogen abundance. The cause for the MAG underrepresentation in some samples remains currently unclear, but this finding illustrates the importance of using a polyphasic sequencing approach to characterize the methanogen community.

**FIG 2:**
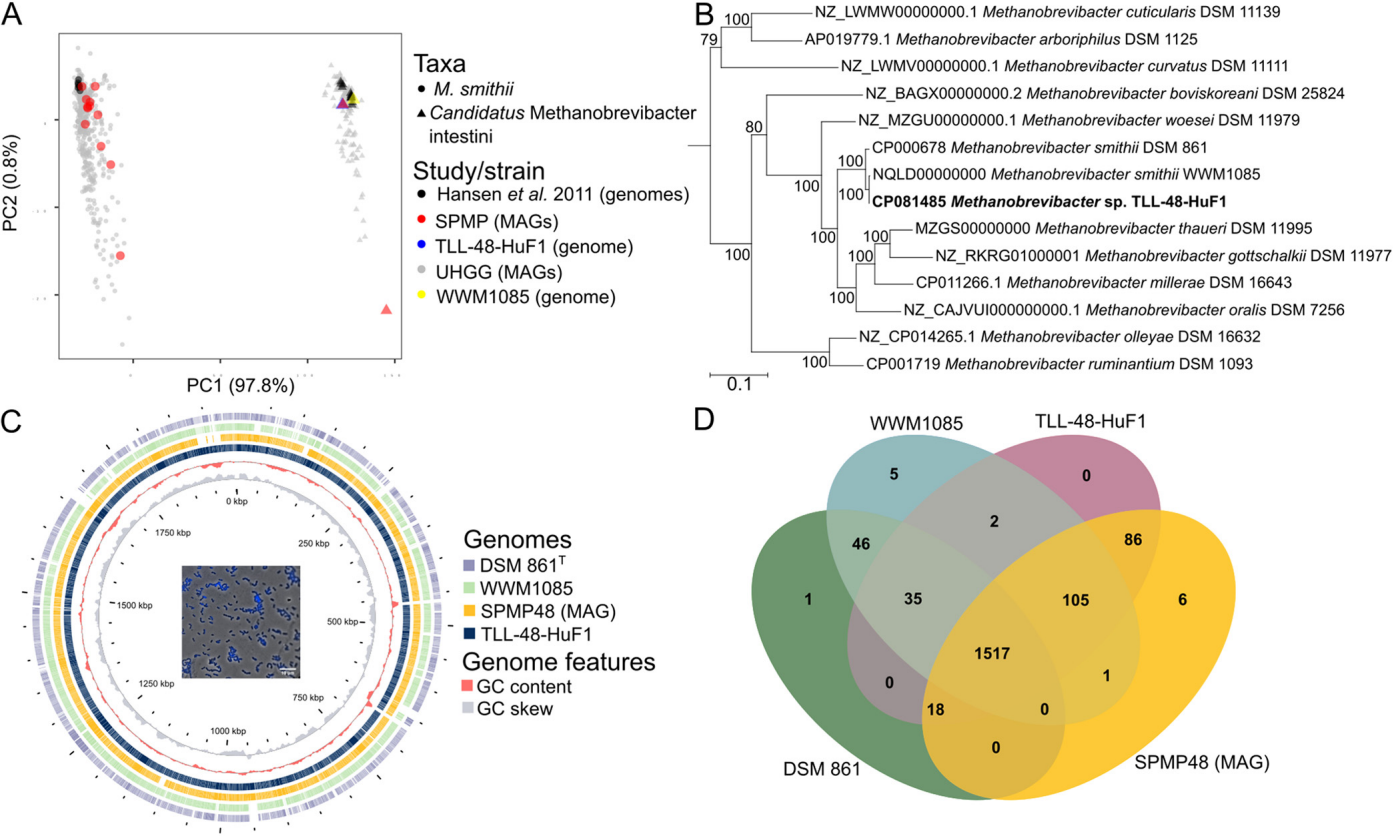
Genomic and phylogenetic analysis of *Candidatus* Methanobrevibacter intestini and *Methanobrevibacter* strains. (A) A principal coordinate analysis plot of average nucleotide identities among *Candidatus* Methanobrevibacter intestini and *Methanobrevibacter* genomes. The variance for each principal coordinate (PC) is shown in parenthesis. The number of MAGs or genomes for each study is *n *= 729 Unified Human Gastrointestinal Genome (UHGG) collection (43), *n *= 14 Singapore Platinum Metagenomes Project (SPMP), *n *= 20 Hansen et al. (5), *n *= 1 TLL-48-HuF1 (this study) and *n *= 1 (WWM1085) (12). (B) A protein tree of concatenated alignment of 43 translated CDS. The tree consists of genomes of *Methanobrevibacter* type strains except for strains WWM1085 and TLL-48-HuF1 as representative strains of *Candidatus* Methanobrevibacter intestini. NCBI GenBank/RefSeq accession number for each taxon are shown. The tree is rooted to *Saccharolobus solfataricus* P1 (NCBI RefSeq NZ_LT549890) and is not shown. Scale bar represents 0.1 substitution per amino acid position. (C) Circular genomic map comparing strain TLL-48-HuF1 reference genome (complete) to metagenome-assembled genome of SPMP48 (incomplete), draft genome of strain WWM1085 and a complete genome of Methanobrevibacter smithii DSM 861^T^. Each ring shows the region of similarity (≥80%) to the reference genome detected by BLAST between translated CDS. CDS were predicted using RAST prior to genomic mapping. The insert is a merged fluorescence and phase contrast image of strain TLL-48-HuF1 viewed at 100 × magnification where blue cells indicate the presence of F420-autoflourescence. Scale bar represents 10 μm. (D) A Venn diagram showing the number of shared orthologous proteins among TLL-48-HuF1, metagenomics assembled genome (MAG) of SPMP48, Methanobrevibacter smithii DSM 861^T^ and *Ca.* Methanobrevibacter intestini WWM1085.

Recent reports suggest that the taxon, Methanobrevibacter smithii may comprise two species (7, 8). Metagenomic databases such as MGnify (9) and Genome Taxonomy Database (10) classified the M. smithii related sequence type as Methanobrevibacter_A smithii_A. A recent analysis of archaeal MAGs purports that M.smithii_A and M. smithii genomes are sufficiently distinct to meet the average nucleotide identity (ANI) species cut-off (>95%) for the former to be classified as ‘*Candidatus* Methanobrevibacter intestini’ (7, 11) (see Table S4 for ANI). We resolved the identities of previously isolated M. smithii strains to show that 13 out of the 20 isolates were *Ca.* Methanobrevibacter intestini strains (Fig. 2A) (5).

The 16S rRNA genes of *Ca.* Methanobrevibacter intestini strains and M. smithii DSM 861^T^ have been previously described as indistinguishable as they share a high sequence identity of >99% (7). Despite their close 16S rRNA gene identity, a few differences exist with the majority found in the V7 region. Specifically, a thymine to cytosine substitution at M. smithii DSM 861^T^ position 1,056 (E. coli position 1,119) and deletions of one to two bases in a homopolymeric thymine-stretch at M. smithii DSM 861^T^ positions 1,076 to 1,081 (E. coli position 1,135 to 1,138) can be observed in the *Ca.* Methanobrevibacter intestini 16S rRNA gene (see Table S3A for all the nucleotide differences). These differences were also detected in *Ca.* Methanobrevibacter intestini strain WWM1085 and 12 isolates from a pan-genomic study of gut M. smithii of related individuals (5, 12) (see Table S3B for nucleotide differences between *Ca*. Methanobrevibacter intestini strains). Due to these differences in the 16S rRNA gene, primer pair Ar915F/Ar1386R (13), which targets the V6-V8 region of the archaeal 16S rRNA gene, was used to characterize methanogen diversity and to distinguish between M. smithii and *Ca.* Methanobrevibacter intestini using AS.

Analysis of amplicon sequences confirmed the presence of *Ca.* Methanobrevibacter intestini in the two MGS samples concomitant with the apparent absence of other methanogen phylotypes (Fig. 1B). This contrasts with 26 samples that were solely of M. smithii phylotype. Co-existence of M. smithii and *Ca.* Methanobrevibacter intestini was detected in seven samples with the latter as a minor component of the methanogen community. In M. smithii positive samples (*n *= 64) the mean relative abundance was 84.1 ± 31.8%. Methanosphaera stadtmanae was detected in eight samples and had a mean relative abundance of 78% ± 17% in three samples that it dominated (Fig. 1B). The low abundance of *Candidatus* Methanobrevibacter intestini may have decreased the likelihood of obtaining more MAGs of at least medium quality from more samples. Overall, AS detected methanogens in 66 of 109 samples (60.5%) (see Fig. S2 for phylotypes in AS samples). Twenty-two out of the 66 samples had methanogen abundance below qPCR detection threshold of 30 copies/μL of sample. In contrast to MGS, these samples revealed the presence of methanogen species and orders, such as Methanomassilicoccales, of which some have been isolated from the human gut (14, 15). Methanogen abundance did not significantly differ among ethnicity (*P = *0.471; Kruskal-Wallis) or gender (false discovery rate adjusted *P = *0.29; Wilcoxon-rank sum test). No significant correlation between methanogen abundance with age was observed (rho = 0.027, *p* = 0.861, Spearman’s rank).

The apparent mutual exclusion of M. smithii and *Ca.* Methanobrevibacter intestini allowed the identification of two samples (SPMP39 and SPMP48) suitable as inoculum for the isolation of a *Ca.* Methanobrevibacter intestini strain. Strain TLL-48-HuF1 (a detailed description of the isolation is provided in the accompanying Materials and Methods section), was successful for one of the samples. Identification of the strain was confirmed by Sanger sequencing the 16S rRNA gene (99.8% of 1,105 bp identity to SPMP48; GenBank accession number OM535902) and the methyl-coenzyme reductase subunit A, *mcrA* gene (100% of 1,406 bp nucleotide identity to SPMP48; GenBank accession number OM642115). McrA and multilocus markers phylogenetic trees show a clear phylogenetic separation between *Ca.* Methanobrevibacter intestini and M. smithii DSM 861^T^ compared to a 16S rRNA gene-based tree (Fig. 2B; Fig. S1) and that *mcrA* may be a more suitable marker gene to analyze methanogen and specifically *Methanobrevibacter* diversity in the gut.

Comparative genome analysis reveals differences between M. smithii and TLL-48-HuF1 (see Fig. 2D; Tables S5, S6, S7) but also a high degree of shared genome content. Notable exceptions include the absence of methyl-coenzyme M reductase isoenzymes (*mrtABDG*), some adhesins-like proteins (ALPs), molybdate transporter genes (*modA* and *modB*), with the latter two also being reported in other *Ca.* Methanobrevibacter intestini genomes (7). The lack of a molybdate transporter could potentially indicate a deficiency of molybdopterin metabolism in the cells, affecting enzymes involved in hydrogenotrophic methanogenesis, such as formyl-methanofuran dehydrogenases. Similar observations have been made for *Msp. stadtmanae*, which is deficient in molybdopterin cofactor (Moco) biosynthesis and thereby restricted to growth on hydrogen and methanol (16). Analysis of the TLL-48-HuF1 genome indicates that it encodes Moco biosynthesis genes and molybdopterin dependent enzyme. In addition, M. smithii DSM 861^T^ and TLL-48-HuF1, showed very similar overall growth characteristics using hydrogen and carbon dioxide for growth (Table S8). While it cannot be completely ruled out that the gene annotation pipelines incorrectly annotated the transporter genes in independent studies, it could also indicate the presence of an alternative, yet to be characterized, molybdate transporter.

The absence of methyl-coenzyme M reductase isoenzyme (*mrtABDG*) as well as differences in ALP repertoire could hint to specific niche adaptations of *Candidatus* Methanobrevibacter intestini. Previous studies have shown that the gene expression of *mcr* and *mrt* genes may be regulated by hydrogen partial pressure (17). However, this has not been investigated in gut environments and genomes of gut methanogens. Adhesin-like proteins, which were originally discovered in the genome of *Msp. stadtmanae*, would potentially also contribute to niche adaptations as has been suggested before (16, 18). However, functional characterizations of ALPs remain poor and hypotheses regarding the purpose of the ALP repertoire *in vivo* remain speculative.

In summary, this study provides insights into the diversity of methanogenic Archaea in the human fecal microbiota of a tri-ethnic cohort in Southeast Asia. The analysis of the relative and absolute abundance of methanogens indicates mutual exclusion of the two species in most samples, which facilitated the isolation of a representative of *Ca.* Methanobrevibacter intestini. Additional phenotypic characterization of *Ca.* Methanobrevibacter intestini is required to determine if it is a novel species or a variant, e.g., a distant sequence type, of M. smithii. The ecological relationship of the two species and the temporal duration of the dominance of one sequence type within a subject remain to be elucidated. It is currently not clear if the dominance of *Ca.* Methanobrevibacter intestini in some subjects is temporary or if the abundance of either species may fluctuate over time. Reanalyzing data from a previous study indicates that heritability may also contribute to the distribution of either species among subjects (5), but this will need to be investigated in more detail with larger cohorts of twins.

## MATERIALS AND METHODS

### Sample collection and DNA extraction.

Feces from 109 individuals aged 48 to 76 years old of the Singapore Integrative Omics Study (SPMP) were collected in 2018 using a BioCollector (BioCollective) kit, according to the manufacturer’s instructions. Fecal samples were handled in a Coy anerobic chamber containing N_2_ (75%), CO_2_ (20%), and H_2_ (5%) gas mixture. Homogenized samples were transferred to 50 mL screw-cap tubes prior to storage at –80°C. The QIAamp Power Fecal Pro DNA kit was used to extract gDNA for genome (2 × 2 mL pure culture; OD_600_ = 0.17), metagenome (fecal material; ~0.5 g) sequencing, quantitative PCR, and 16S amplicon sequencing. DNA for genomic sequencing was further purified using a Qiagen Genomic Tip 20/g kit as described in the manufacturer’s protocol (Qiagen, Germany). Cells from cultures were concentrated at 10,000 × *g* for 15 min before DNA extraction. DNA was quantified using a Qubit 1.0 fluorometer with a broad range assay kit (Life Technologies) and a NanoDrop-2000 (Thermo Fisher Scientific).

### Single marker gene-based analyses.

Using general primers for amplification of bacterial 16S rRNA genes and strain TLL-48-HuF1 *mcrA*-specific primers were used to test culture purity and enrichment of methanogen, respectively. Primer sequences and annealing temperatures are stated in Table S9. Each PCR contained 1 × GoTaq master mix (Promega), 0.2 μM final concentration per primer, and 1.7 ng/μL template. Thermal-cycler condition was generally 95°C for 3 min, 32 cycles of 95°C for 30 s, annealing temperature (Table S9) for 30 s, and 72°C for 30 s (amplicon ≤ 500 bp) followed by a final extension of 72°C for 10 min, unless stated otherwise. Primers designed in this study were checked for specificity using PRIMER-BLAST against NCBI nonredundant nucleotide database using default parameters (19). PCR products were separated by gel electrophoresis using 1.5% (≥1 kb) or 2% (<1 kb) agarose gel in 1 × TAE buffer (40 mM Tris, 20 mM acetic acid, 1 mM EDTA) stained with 1 × FloroSafe (1st Base). Primers Ar915F/Ar1386R were used to quantify methanogenic Archaeal 16S rRNA genes with SYBR-green based qPCR chemistry as previously described (13). The average quantity (copies/μL) of triplicate reactions was quantified as technical replicates and normalized by the amount (ng) of DNA extracted per sample. Standards used for qPCR were 10-fold dilutions of pGEM-T Easy vectors (Promega) ligated with M. smithii DSM 861^T^ 16S rRNA gene 1.3 kb amplicons, which were amplified using Ar84F/Ar1386R. The standard curve ranged from 3 × 10^1 to 8^ copies/μL of sample, with a slope of −3.6, y-intercept of 34.8, and R^2^ = 0.97. Postamplification melt curve analysis was used to check for reaction specificity based on the average melting temperature (Tm) of the 16S rRNA gene standard (87 ± 0.2°C). Primer pair mcrA-85F/mcrA-1649R was used to amplify a 1.5 kb fragment of the *mcrA* gene for Sangar sequencing.

### Cultivation and isolation of strains.

Methanobrevibacter smithii PS (DSM 861^T^ = ATCC 35061^T^) was purchased from Deutsche Sammlung von Mikroorganismen und Zellkulturen GmbH. Cultures were grown as batch cultures contained in 100 mL serum bottles with 20 mL sterile basal medium enclosed with butyl rubber stoppers and aluminum crimp caps, unless stated otherwise. Basal medium was also used to prepare semisolid agarose shakes (0.7% low melting agarose; Life Technologies) and 3% noble agar. The basal medium composition followed medium 1 described in Balch et al. (20) with less Trypticase peptone and yeast extract (1 g/L each) added. Na_2_S.9H_2_O 0.25% (wt/vol) and 1 g/L l-cysteine-HCl were added as reducing agents. Sodium bicarbonate 10% (wt/vol) and HEPES (1g/L) buffered the medium at pH 7. All chemicals were purchased from Sigma-Aldrich unless stated otherwise. Cultures were pressurized with H_2_:CO_2_ (4:1) at 1.8 bar unless stated otherwise. All cultures were incubated in the dark at 37°C with liquid cultures shaken horizontally at 150 rpm. Gases except hydrogen were purchased from Air Liquide (Singapore) at 99.9995% minimum purity. Hydrogen was generated using a LNI SwissGas HG PRO (Italy) hydrogen generator at 99.9999% purity. Growth was monitored spectrophotometrically at 600 nm optical density (OD_600_) in an Amersham Biosciences Ultrospec 2100 pro. For enrichment culture, 0.6 g (wet weight) fecal material from a 71-year-old (SGM-48) was added to basal medium amended with ampicillin (100 μg/mL), tetracycline (10 μg/mL), and vancomycin (25 μg/mL) to inhibit bacterial growth. After three consecutive weekly transfers (10%; vol/vol) of the parent enrichment culture, additional ampicillin (200 μg/mL), tetracycline (35 μg/mL), vancomycin (50 μg/mL), and norfloxacin (10 μg/mL) were added to a fourth transfer. Rumen fluid (5% vol/vol) and 0.5 g/L coenzyme M were also added to provide nutrients that might be lacking from the transfers. After 6 days of incubation, serial dilutions (10^−1 to −9^) and semisolid agarose shakes (0.7% wt/vol; Life Technologies) were prepared in 10-mL headspace glass vials (Agilent Technologies) filled with 3 mL basal medium. Colonies were picked using 1-mL syringes with 21 G 1.5-inch hypodermic needles (BD) and inoculated as batch cultures. To ensure purity, strain TLL-48-HuF1 was further streaked onto 3% (wt/vol) noble agar basal medium (12 mL) contained in agar bottle plates (Bellco Glass) with 1 bar of H_2_:CO_2_ (4:1). Substrate utilization tests were performed in triplicate batch cultures in basal media devoid of rumen fluid that contained sodium acetate (61 mM), sodium formate (74 mM), methanol (123.6 mM), or trimethylamine (52 mM). Cultures without hydrogen were filled with N_2_:CO_2_ (4:1) at 1.8 bar. OD_600_ was measured to monitor for growth.

### Phase contrast and fluorescence microscopy.

Cultures (OD_600_ = ~0.4) were immobilized on 2% (wt/vol) agarose coated slides and visualized on an inverted microscope (Zeiss Axio Observer 7) equipped with a 100×/1.4 plan apochromat objective lens, a fluorescence filter (BP 365/12 excitation and LP 397 emission), and a Hamamatsu 2k × 2k CMOS camera. Phase contrast and fluorescent images were merged, and scale bars added using MetaMorph version 7.10.2.240 (Molecular Devices LLC). ImageJ version 1.53e was used to obtain the average length of 100 cells from five fields (21).

### Methane analysis.

Methane was measured from culture headspace using a gas chromatograph equipped with a flame ionization detector (GC-FID; Agilent Technologies 6890). Headspace (50 μL) was manually injected using a 250 μL lockable gas-tight syringe (Trajan Scientific and Medical) into the GC-FID. The GC was equipped with a 30 m × 0.32 mm internal diameter GasPro columns (J&W Scientific). Methane was quantified from calibration curves of five-point standards (10 μmol, 100 μmol, 1 mmol, 10 mmol, 100 mmol) that were prepared in bottles under the same conditions as the batch cultures.

### Sequencing library construction and DNA sequencing.

Library construct for 16S rRNA gene Illumina amplicon sequencing protocol followed that described for the bacterial 16S rRNA gene (22). The barcoded bacterial 515F/806R primers were replaced with methanogen specific primers Ar915F/Ar1386R (Table S9) while retaining the same sequences for Illumina adapters, Golay barcodes, primer pads, and linkers. PCR followed a previously described condition (13). The library preparation was sequenced on an Illumina MiSeq using 2 × 250 bp chemistry by an external vendor (Axil Scientific). The demultiplexed raw fastq reads were processed using Qiime 2 2021.4 release (23). Forward reads were denoised using DeBlur to obtain amplicon sequence variants (ASV) (24). ASVs fewer than four reads in total were removed to minimize spurious reads (25). ASVs were BLASTed against RIM-DB, a database specific to methanogens of human and animal gut at 80% identity threshold (26). The proportion of ASV per sample is based on a rarefied depth of 1,220 reads per sample using the “qiime diversity core-metrics-phylogenetic” command and default options. Full-length 16S rRNA gene sequence from SPMP48 (MAG) was manually added to RIM-DB prior to clustering at 99.5% using vsearch to remove identical sequences (27). The genome of strain TLL-48-HuF1 was sequenced on a Illumina NovaSeq 6000 Sequencing System and an Oxford Nanopore Technologies MinION equipped with a R9.4.1 flow cell to sequencing depths of 640 × and 208 ×, respectively. Library preparation for paired-end sequencing (2 × 150 bp) on NovaSeq PE150 flow cell was performed externally (NovogeneAIT). MinION sequencing for isolate genome and MAGs was performed using a ligation sequencing kit (SQK-LSK109) and base called using MinIT (Oxford Nanopore Technologies PLC) according to the manufacturer’s protocol. A hybrid genome assembly utilizing short and long reads was generated assembled using UniCycler version 0.4.8 using default parameters (28) (see Table S10 for sequencing coverage and statistics). The SPMP metagenomic library for short reads was constructed using NEBNext Ultra II FS DNA Library Prep Kit for Illumina (New England Biolabs) and paired-end sequenced (2 × 151 bp reads) on an Illumina HiSeq4K platform as previously described (29).

### Comparative genome analysis.

Protein coding sequences (CDS) were predicted and annotated from genomes using RAST server (30). Genome statistics was calculated NCBI Prokaryote Annotation Pipeline (31). Circular genomes of CDS were generated using Gview using default parameters (32). Venn diagrams and Swiss-Prot annotations were obtained using OrthoVenn2 (e-value cutoff = 0.01) (33). BlastKoala was used to provide annotation against the Kyoto encyclopedia of genes and genomes (KEGG) database (34). FastANI v1.33 was used calculate ANI between genomes (11).

### Phylogenetic analyses.

Maximum likelihood phylogenetic trees from 16S rRNA gene and McrA were generated using raxmlGUI 2.0.5 (35) and bootstrapped using 1,000 iterations each. CheckM v1.1.2 was used to obtain a concatenated amino acid sequence of 43 CDS from each of the 15 genomes compared, including strain WWM1085 (12, 36). A maximum likelihood tree from the 43 CDS was constructed using RAxML v7.0.3 based on the PROTGAMMAJTTF model and 1,000 bootstrap iterations (37).

### Metagenomic sequencing assembly and analysis.

Methanogen MAGs are derived from the Singapore Platinum Metagenomes Project (SPMP) (29). Methanogen MAGs are hybrid assemblies of Illumina and MinION data using OPERA-MS v0.9.0 (38). Representative MAGs binned at species level cluster (ANI >95%, mash distances [39]) using sklearn v0.23.2 (40) were assigned taxonomic identities using MAG databases of GTDB-Tk v1.4.1 to Genome Taxonomic Database (GTDB; release 95) (41, 42), and Unified Human Gastrointestinal Genome (UHGG) (43). Relative abundances for MGS data were calculated using Kraken and Bracken with a custom database generated from MGS MAGs (44, 45). Genome quality was assessed for completeness and contamination using CheckM v1.04 (36), Trna and rRNA content using tRNA-scan SE v2.0.5 (46), and barrnap v0.9 (https://github.com/tseemann/barrnap) where threshold for quality MAGs followed the minimum information about a metagenome-assembled genome guidelines (47). MAGs from UHGG collection were downloaded from Mgnify (9). The principal coordinate analysis plot was drawn using the ggplot2 (48) package in R v.4.1.2 (49) based on pairwise fastANI v1.32 (11) distances between a set of genomes composed of both MGS MAGs and external genomes, including UHGG genomes with ANI > 95% (mash distances) and manually selected genomes.

### NCBI accession numbers.

Raw AS fastq files have been deposited under BioProject number PRJNA780363. 16S rRNA gene and *mcrA* sequences from this study were deposited to GenBank with the respective accession numbers OM535902 and OM642115. The hybrid genome of strain TLL-48-HuF1 is assigned GenBank accession number CP081485. MGS short and long reads can be found under BioProject number PRJEB49168. MAGs are deposited at https://zenodo.org/record/6537609#.YnsqD4xByUk.
