# Supplementary material for: Mutual Exclusion of Methanobrevibacter Species in the Human Gut Microbiota Facilitates Directed Cultivation of a Candidatus Methanobrevibacter Intestini Representative
Source: Microbiol Spectr. 2022 Jun 14;10(4):e00849-22. doi: 10.1128/spectrum.00849-22 (PMC9431525; doi:10.1128/spectrum.00849-22)
Supplement: Supplemental file 1 — Supplemental material. Download spectrum.00849-22-s0001.pdf, PDF file, 3.9 MB [file spectrum.00849-22-s0001.pdf]

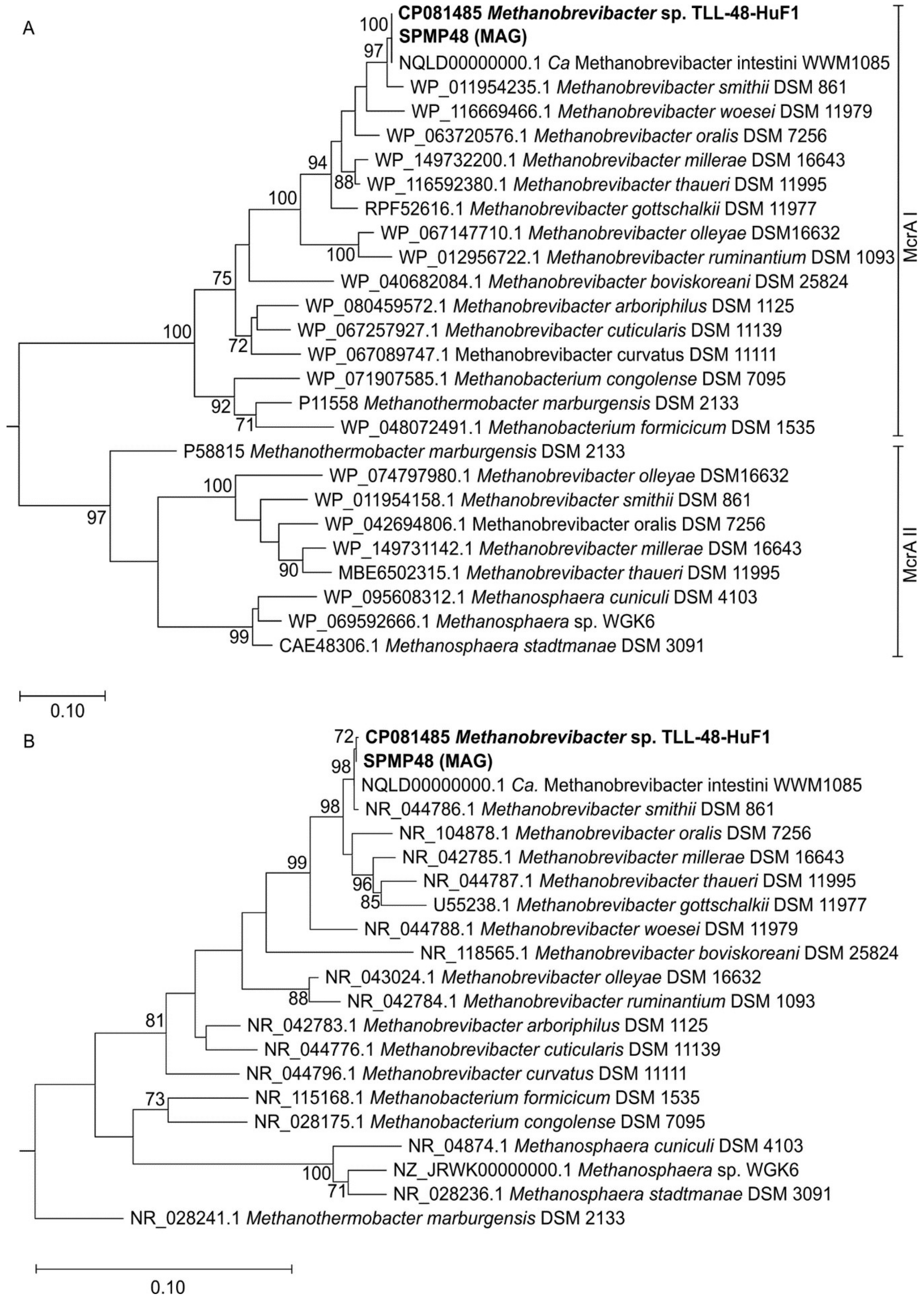

FIG S1 Phlyogenetic trees comparing strain TLL-48-HuF1 to *Methanobrevibacter* type strains and *Candidatus* Methanobrevibacter intestini representative strain WWM1085. (A) A maximum

likelihood phylogenetic tree of McrA protein sequences of type strains of the Methanobacteriales order including *Methanobrevibacter smithii* WWM1085 of a human fecal isolate and *Methanosphaera* sp. WGK6 a gut isolate of a Western grey kangaroo. Some strains harbor the methyl-coenzyme M reductase isoenzyme, which forms a clade denoted by McrA II. McrA of Candidatus Bathyarchaeota archaeon BA1 (KPV65186.1) and Candidatus Bathyarchaeota archaeon BA2 (KPV61791.1) were used as outgroups and not shown. (B) A maximum likelihood phylogenetic tree of 16S rRNA genes of the same strains as the McrA tree with exception to *Saccharolobus solfataricus* strain DSM 1616 (NR_119198.1) as an outgroup. NCBI (GenBank/RefSeq) accession numbers come afore taxonomic nomenclatures. Sequences from this study are highlighted in bold. Bootstrap values for both trees were generated using 1,000 iterations. Only bootstrap values above 70% are shown. Scale bar for branch length indicate 0.10 average nucleotide or amino acid change per position.

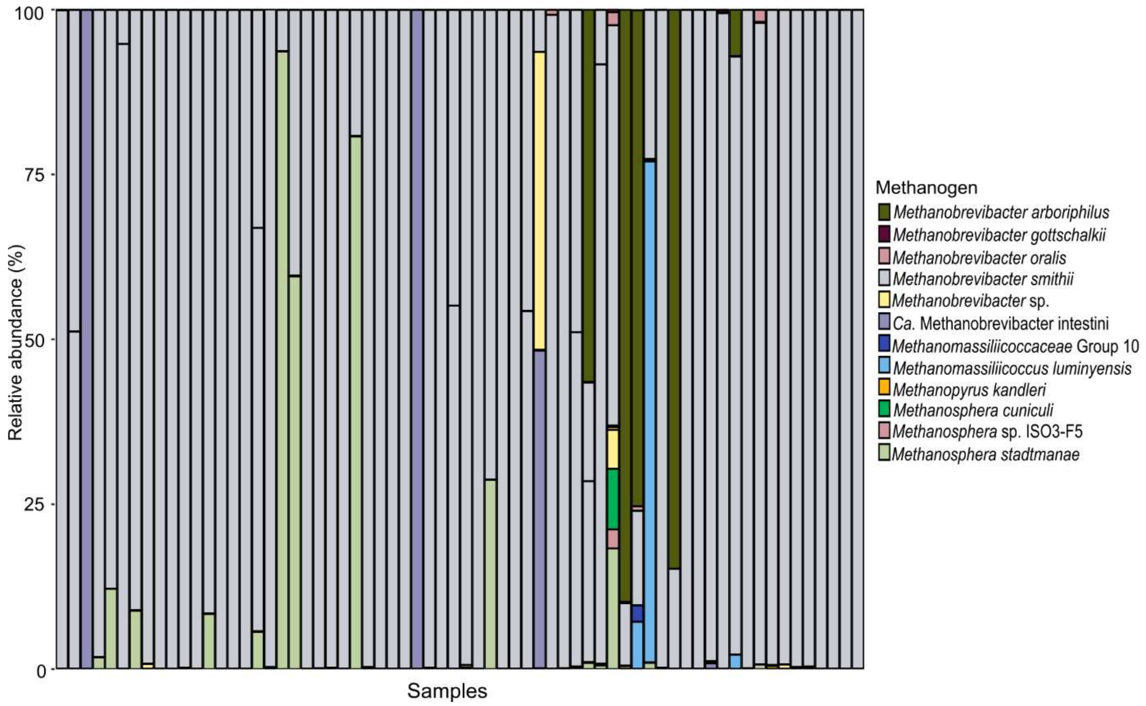

FIG S2 A stacked barplot of methanogens in adult fecal samples (*n*=66) based on 16S amplicon sequencing using Ar915F/Ar1386R primers. The samples are arranged from highest to lowest (left to right) 16S rRNA gene copies/ng of DNA. Sequences were BLAST against RIM-DB database clustered at 95% sequenced identity.

Table S1. Metadata for Singapore Platinum Metagenomes Project (SPMP).

Sample ID	Sample Type	Nationality	Age	Gender	Race
SPMP1	Stool	Singaporean	57	M	Indian
SPMP2	Stool	Singaporean	52	F	Indian
SPMP3	Stool	Singaporean	52	F	Indian
SPMP4	Stool	Singaporean	48	M	Indian
SPMP5	Stool	Singaporean	54	F	Malay
SPMP6	Stool	Singaporean	68	M	Malay
SPMP7	Stool	Singaporean	61	F	Malay
SPMP8	Stool	Singaporean	54	M	Malay
SPMP9	Stool	Singaporean	59	M	Indian
SPMP10	Stool	Singaporean	51	F	Malay
SPMP11	Stool	Singaporean	51	M	Indian
SPMP12	Stool	Singaporean	58	F	Malay
SPMP13	Stool	Singaporean	60	F	Malay
SPMP14	Stool	Singaporean	67	F	Indian
SPMP15	Stool	Singaporean	71	F	Malay
SPMP16	Stool	Singaporean	68	F	Indian
SPMP17	Stool	Singaporean	56	F	Malay
SPMP18	Stool	Singaporean	58	F	Chinese
SPMP19	Stool	Singaporean	56	F	Chinese
SPMP20	Stool	Singaporean	60	F	Chinese
SPMP21	Stool	Singaporean	69	F	Chinese
SPMP22	Stool	Singaporean	59	F	Chinese
SPMP23	Stool	Singaporean	62	F	Indian
SPMP24	Stool	Singaporean	65	F	Indian
SPMP25	Stool	Singaporean	66	M	Indian
SPMP26	Stool	Singaporean	60	M	Indian
SPMP27	Stool	Singaporean	56	F	Chinese
SPMP28	Stool	Singaporean	68	M	Indian
SPMP29	Stool	Singaporean	56	F	Indian
SPMP30	Stool	Singaporean	57	M	Chinese
SPMP31	Stool	Singaporean	64	M	Chinese
SPMP32	Stool	Singaporean	60	F	Indian
SPMP33	Stool	Singaporean	59	M	Chinese
SPMP34	Stool	Singaporean	65	M	Chinese
SPMP35	Stool	Singaporean	71	F	Indian
SPMP36	Stool	Singaporean	53	M	Chinese
SPMP37	Stool	Singaporean	73	M	Chinese
SPMP38	Stool	Singaporean	62	F	Indian
SPMP39	Stool	Singaporean	60	F	Chinese
SPMP40	Stool	Singaporean	58	F	Chinese
SPMP41	Stool	Singaporean	50	M	Indian
SPMP42	Stool	Singaporean	60	F	Chinese
SPMP43	Stool	Singaporean	63	M	Chinese
SPMP44	Stool	Singaporean	57	F	Chinese
SPMP45	Stool	Singaporean	61	F	Indian
SPMP46	Stool	Singaporean	62	F	Chinese
SPMP47	Stool	Singaporean	65	F	Indian
SPMP48	Stool	Singaporean	71	F	Malay
SPMP49	Stool	Singaporean	63	F	Chinese
SPMP50	Stool	Singaporean	67	M	Chinese
SPMP51	Stool	Singaporean	59	M	Indian
SPMP52	Stool	Singaporean	61	F	Chinese
SPMP53	Stool	Singaporean	63	F	Chinese
SPMP54	Stool	Singaporean	67	F	Chinese
SPMP55	Stool	Singaporean	58	F	Chinese
SPMP56	Stool	Singaporean	68	F	Chinese
SPMP57	Stool	Singaporean	62	M	Chinese
SPMP58	Stool	Singaporean	60	F	Chinese
SPMP59	Stool	Singaporean	62	F	Chinese
SPMP60	Stool	Singaporean	50	M	Malay
SPMP61	Stool	Singaporean	61	M	Chinese
SPMP62	Stool	Singaporean	57	F	Malay
SPMP63	Stool	Singaporean	58	F	Chinese
SPMP64	Stool	Singaporean	60	M	Malay
SPMP65	Stool	Singaporean	60	M	Chinese
SPMP66	Stool	Singaporean	55	F	Chinese
SPMP67	Stool	Singaporean	60	F	Chinese
SPMP68	Stool	Singaporean	52	M	Malay
SPMP69	Stool	Singaporean	52	F	Malay
SPMP70	Stool	Singaporean	57	M	Malay
SPMP71	Stool	Singaporean	51	F	Malay
SPMP72	Stool	Singaporean	62	F	Indian
SPMP73	Stool	Singaporean	48	F	Malay
SPMP74	Stool	Singaporean	55	M	Chinese
SPMP75	Stool	Singaporean	53	M	Chinese
SPMP76	Stool	Singaporean	57	M	Malay
SPMP77	Stool	Singaporean	62	M	Chinese
SPMP78	Stool	Singaporean	58	F	Malay
SPMP79	Stool	Singaporean	56	F	Malay
SPMP80	Stool	Singaporean	49	F	Malay
SPMP81	Stool	Singaporean	52	F	Indian
SPMP82	Stool	Singaporean	67	M	Indian
SPMP83	Stool	Singaporean	50	M	Malay
SPMP84	Stool	Singaporean	61	M	Chinese
SPMP85	Stool	Singaporean	57	F	Chinese
SPMP86	Stool	Singaporean	60	F	Malay
SPMP87	Stool	Singaporean	61	F	Chinese
SPMP88	Stool	Singaporean	61	F	Chinese
SPMP89	Stool	Singaporean	62	F	Indian
SPMP90	Stool	Singaporean	60	F	Chinese
SPMP91	Stool	Singaporean	64	F	Chinese
SPMP92	Stool	Singaporean	66	F	Malay
SPMP93	Stool	Singaporean	53	M	Indian
SPMP94	Stool	Singaporean	59	M	Chinese
SPMP95	Stool	Singaporean	67	M	Chinese
SPMP96	Stool	Singaporean	74	F	Chinese
SPMP97	Stool	Singaporean	54	M	Malay
SPMP98	Stool	Singaporean	54	M	Chinese
SPMP99	Stool	Singaporean	60	M	Chinese
SPMP100	Stool	Singaporean	59	F	Chinese
SPMP101	Stool	Singaporean	60	M	Chinese
SPMP102	Stool	Singaporean	56	F	Chinese
SPMP103	Stool	Singaporean	62	M	Indian
SPMP104	Stool	Singaporean	57	F	Indian
SPMP105	Stool	Singaporean	59	M	Chinese
SPMP106	Stool	Singaporean	56	F	Chinese
SPMP107	Stool	Singaporean	76	M	Chinese
SPMP108	Stool	Singaporean	49	M	Indian
SPMP109	Stool	Singaporean	66	F	Indian

Table S2. Metagenomic assembled genome (MAG) statistics and quality.

MAG Sample ID	Species	Size (bp)	#_contigs	Contig_N50	Longest_contig	Completeness (	Contamination	Species Level Cluster (SLC)	MIMAG (NC: Near complete)
SPMP03_bin.52	Methanobrevibacter_A smithii	1377976	69	31168	125079	56.29	0.31	159	MEDIUM
SPMP04_bin.116	Methanobrevibacter_A smithii	1601359	46	72088	140159	79.73	0	159	MEDIUM
SPMP05_bin.17	Methanobrevibacter_A smithii	1833101	2	1763845	1763845	99.2	1.6	159	NC
SPMP05_bin.41	Methanosphaera stadtmanae	1699540	27	96629	279033	86.14	0.8	416	MEDIUM
SPMP13_bin.9	Methanobrevibacter_A smithii	1792840	3	1782837	1782837	98	0.8	159	NC
SPMP18_bin.5	Methanobrevibacter_A smithii	1801357	2	1278871	1278871	99.2	0	159	NC
SPMP25_bin.80	Methanobrevibacter_A smithii	1635828	35	88240	299168	83.39	0	159	MEDIUM
SPMP26_bin.4	Methanosphaera stadtmanae	1692852	4	641713	681328	96.8	0	416	HIGH
SPMP26_bin.8	Methanobrevibacter_A smithii	1763862	5	1009593	1009593	99.16	0	159	NC
SPMP30_bin.136	Methanobrevibacter_A smithii	2798878	103	1758261	1758261	100	0	159	NC
SPMP30_bin.148	Methanosphaera stadtmanae	1468228	59	68092	159469	77.81	2.4	416	MEDIUM
SPMP31_bin.129	Methanobrevibacter_A smithii	1853786	2	1787433	1787433	100	0	159	NC
SPMP31_bin.71	Methanosphaera stadtmanae	1488493	19	122109	266271	75.81	0	416	MEDIUM
SPMP39_bin.88	Methanobrevibacter_A smithii_A	1229812	78	24837	64848	57.58	0	84	MEDIUM
SPMP48_bin.130	Methanobrevibacter_A smithii_A	1901350	3	1639946	1639946	97.14	0.8	84	NC
SPMP51_bin.133	Methanobrevibacter_A smithii	1750191	4	942232	942232	97.6	0	159	NC
SPMP88_bin.19	Methanobrevibacter_A smithii	1846791	135	29285	83533	90.57	0.8	159	NC
SPMP102_bin.110	Methanosphaera stadtmanae	1830480	9	451050	720204	96.8	0	416	HIGH
SPMP102_bin.4	Methanobrevibacter_A smithii	1744806	7	246028	697833	99.2	0	159	NC

Table S3A. Nucleotide differences between 16S rRNA genes of Methanobrevibacter smithii DSM 861[T] and Candidatus Methanobrevibacter intestini (SPMP48)

	72*[a]	268[b]	376[b]	451[c]	1056[d]	1080[d]	1081[d]	1394[e]
Methanobrevibacter smithii DSM 861[T] (GenBank accession number NC_009515; 1478 bp)	G	C	G	C	T	T	T	T
SPMP48 (MAG; 1475 bp)	A	T/C^	-	T	C	-	-	G

*Positions are based on the 16S rRNA gene of *M. smithii* DSM 861[T] (GenBank accession number NC_009515).

[a]V1 region, [b]V3 region, [c]V4 region, [d] V7 region, [e] V9 region

^Cytosine was obtained from Sangar sequencing

Table S3B. Nucleotide differences between 16S rRNA genes of Methanobrevibacter smithii WWM1085 and Methanobrevibacter sp. TLL-48-HuF1.

	268*[a]	451[b]	1074[c]
TLL-48-HuF1 (NCBI CP081485; 1475 bp)	T/C^	T	C
WWM1085 (NCBI RefSeq NZ_NQLD01000012.1; 1475 bp)	C	C	T/C

*Positions are based on the 16S rRNA gene of *M. smithii* DSM 861[T] (GenBank accession NC_009515).

[a]V3 region, [b]V4 region, [c]V7 region

^Cytosine was obtained from Sangar sequencing

Table S4. Average nucleotide identity (%) of Methanobrevibacter species.

	M. arboriphilus DSM 1125	*M. boviskoreani* DSM 25824	*M. curvatus* DSM 11111	*M. cuticularis* DSM 11139	*M. gottschalker* DSM 11977	*M. millerae* DSM 16643	*M. olleyae* DSM 16632	*M. oralis* DSM 7256	*M. ruminantium* DSM 1093	*M. smithii* DSM 861	*Candidatus* Methanobrevibacter intestini WWM1085	*M. thaueri* DSM 11995	*M. woesei* DSM 11979
M. arboriphilus DSM 1125													
M. boviskoreani DSM 25824	<75												
M. curvatus DSM 11111	76.3	<75											
M. cuticularis DSM 11139	78.3	<75	77.0										
M. gottschalker DSM 11977	76.2	<75	<75	<75									
M. millerae DSM 16643	<75	<75	<75	<75	79								
M. olleyae DSM 16632	76.0	76.7	75.8	76.1	77.9	<75							
M. oralis DSM 7256	76.2	<75	<75	<75	80.1	78.4	77.1						
M. ruminantium DSM 1093	<75	<75	<75	<75	<75	<75	79.9	<75					
M. smithii DSM 861	76.4	<75	<75	<75	79.6	78.9	77.2	79.6	<75				
Candidatus Methanobrevibacter intestini WWM1085	<75	<75	<75	76.3	79.6	79.0	77.5	79.8	<75	93.6			
M. thaueri DSM 11995	<75	<75	<75	<75	80.4	79.5	<75	79.4	<75	79.3	79.4		
M. woesei DSM 11979	76.7	77.9	76.3	77.0	78.9	<75	77.8	79.1	<75	80.2	80.4	79.1	
Methanobrevibacter sp. TLL-48-HuF1	76.5	<75	<75	76.3	79.4	78.9	77.4	79.7	<75	93.7	98.4	79.4	80.2

Table S5. Genome features of Methanobrevibacter smithii DSM 861^T, strains TLL-48-HuF1 and WWM1085.

	TLL-48-HuF1 (CP081485)	*M. smithii* DSM 861^T (CP000678)	*Candidatus* Methanobrevibacter intestini WWM1085 (SAMN07492084)
Last annotation date on NCBI uisng the NCBI Prokaryotic Genome Annotation Pipeline (PGAP)	19-Aug-21	5-Jan-22	19-Jun-21
Total genome length (bp)	1,951,395	1,853,160	1,926,345
DNA GC content (bp)	592,204	575,114	543,228
GC(%)	30.35%	31.03%	30.30%
Total genes	1,840	1,804	1,852
5S rRNA gene	3	3	3
16S rRNA gene	2	2	3^
LSU rRNA genes	2	2	3^
tRNA genes	35	36	35
non-coding RNAs	2	2	2
CRISPR array	0	1	2
Total protein coding seqeunce (CDS)	1,777	1,759	1,753
Pseudogenes	40	22	53
mcrA gene (type)	1 (McrA I)	2 (McrA I and McrA II)	1 (McrA I)

^Two of which are partial genes.

Table S6. SwissProt annotation of unique proteins based on pairwise comparison of TLL-48-HuF1 to other genomes. (Unannotated proteins are not shown)

No.	swiss_prot_id	Gene	Protein
**Proteins unique to TLL-48-HuF1 vs DSM 861^T**			
1	B0NAQ4	baiN	3-dehydro-bile acid delta(4,6)-reductase
2	Q2NB77	ELI_04860	Blue-light-activated histidine kinase 2
3	P20021	cadA	Cadmium-transporting ATPase
4	Q1RKM3	pgsA	CDP-diacylglycerol--glycerol-3-phosphate 3-phosphatidyltransferase
5	Q10717	CCP2	Cysteine proteinase 2
6	P9WJW8	jefA	Drug efflux pump JefA
7	P25148	gspA	General stress protein A
8	B2THR0	gpsA	Glycerol-3-phosphate dehydrogenase [NAD(P)+]
9	Q8XGT8	gdx	Guanidinium exporter
10	Q57603	mvp	Hyperpolarization-activated voltage-gated potassium channel
11	P34877	scrFIAM	Modification methylase ScrFIA
12	P9WGL4	pdtaS	Probable sensor histidine kinase PdtaS
13	P9WGM2	pdtaR	Probable transcriptional regulatory protein PdtaR
14	Q9JWD6	vsr	Putative very short patch repair endonuclease
15	O31423	skfB	Sporulation killing factor maturation protein SkfB
16	O59151	tfb	Transcription initiation factor IIB
17	P10484	hsdM	Type I restriction enzyme EcoR124II M protein
18	Q57594	MJ0130	Type-1 restriction enzyme MjaXIP specificity protein
19	Q5HK76	hsdR	Type-1 restriction enzyme R protein
20	P36433	hpaIIR	Type-2 restriction enzyme HpaII
21	Q4L5V4	Q4L5V4	Tyrosine recombinase XerC
**Proteins unique to TLL-48-HuF1 vs WWM1085**			
1	B0NAQ4	baiN	3-dehydro-bile acid delta(4,6)-reductase
2	Q2NB77	ELI_04860	Blue-light-activated histidine kinase 2
3	Q8TXF9	albA1	DNA/RNA-binding protein Alba 1
4	Q00405	vhcD	F420-non-reducing hydrogenase vhc iron-sulfur subunit D
5	Q60316	fdhB	Formate dehydrogenase subunit beta
6	P26162	bchH	Magnesium-chelatase subunit H
7	P34877	scrFIAM	Modification methylase ScrFIA
8	P60232	mvhB	Polyferredoxin protein MvhB
9	P9WGL4	pdtaS	Probable sensor histidine kinase PdtaS
10	P9WGM2	pdtaR	Probable transcriptional regulatory protein PdtaR
11	Q9JWD6	vsr	Putative very short patch repair endonuclease
12	O31423	skfB	Sporulation killing factor maturation protein SkfB
13	P10484	hsdM	Type I restriction enzyme EcoR124II M protein
14	Q57594	MJ0130	Type-1 restriction enzyme MjaXIP specificity protein
15	Q57594	MJ0130	Type-1 restriction enzyme MjaXIP specificity protein
16	Q5HK76	hsdR	Type-1 restriction enzyme R protein
17	P36433	hpaIIR	Type-2 restriction enzyme HpaII
**Proteins unique to DSM861^T vs TLL-48-HuF1**			
1	Q57831	cas2	CRISPR-associated endoribonuclease Cas2
2	O58931	cas6	CRISPR-associated endoribonuclease Cas6
3	Q57822	cas4	CRISPR-associated exonuclease Cas4
4	D3E288	cas1-1	CRISPR-associated protein Cas1 1
**Proteins unique to WWM1085 vs TLL48HuF1**			
1	P27373	slgA	Cell surface glycoprotein
2	Q57831	cas2	CRISPR-associated endoribonuclease Cas2
3	O58931	cas6	CRISPR-associated endoribonuclease Cas6
4	Q57822	cas4	CRISPR-associated exonuclease Cas4
5	D3E288	cas1-1	CRISPR-associated protein Cas1 1

Table S7. KEGG annotation of unique proteins based on pairwise comparison of TLL-48-HuF1 to other strains. (Unannotated proteins are not shown)

No	KEGG ID	Gene	KO Description
**Proteins unique to TLL-48-HuF1 vs DSM 861^T (Proteins in blue are also not found in TLL-48 (MAG) and WWM1085 genomes)**			
1	K22010	pdtaR	two-component system, response regulator PdtaR
2	K01154	hsdS	type I restriction enzyme, S subunit [EC:3.1.21.3]
3	K03427	hsdM	type I restriction enzyme M protein [EC:2.1.1.72]
4	K01153	hsdR	type I restriction enzyme, R subunit [EC:3.1.21.3]
5	K00558	DNMT1, dcm	DNA (cytosine-5)-methyltransferase 1 [EC:2.1.1.37]
6	K07458	vsr	DNA mismatch endonuclease, patch repair protein [EC:3.1.-.-]
7	K07133	K07133	uncharacterized protein
8	K02107	ATPVG, ahaH, atpH	V/A-type H+/Na+-transporting ATPase subunit G/H
9	K02472	wecC	UDP-N-acetyl-D-mannosaminuronic acid dehydrogenase [EC:1.1.1.336]
10	K07133	K07133	uncharacterized protein
**Proteins unique to TLL-48-HuF1 vs WWM1085**			
1	K22010	pdtaR	two-component system, response regulator PdtaR
2	K01154	hsdS	type I restriction enzyme, S subunit [EC:3.1.21.3]
3	K03427	hsdM	type I restriction enzyme M protein [EC:2.1.1.72]
4	K01153	hsdR	type I restriction enzyme, R subunit [EC:3.1.21.3]
5	K00558	DNMT1, dcm	DNA (cytosine-5)-methyltransferase 1 [EC:2.1.1.37]
6	K07458	vsr	DNA mismatch endonuclease, patch repair protein [EC:3.1.-.-]
7	K07133	K07133	uncharacterized protein
**Proteins unique to SPMP-48 (MAG) vs TLL-48-HuF1 (Proteins in blue are also not found in DSM 861^T and WWM1085 genomes)**			
1	K00077	panE, apbA	2-dehydropantoate 2-reductase [EC:1.1.1.169]
2	K02107	ATPVG, ahaH, atpH	V/A-type H+/Na+-transporting ATPase subunit G/H
**Proteins unique to DSM 861^T vs TLL-48-HuF1 (Proteins in blue are also not found in TLL-48 (MAG) and WWM1085 genomes)**			
1	K07133	K07133	uncharacterized protein
2	K09951	cas2	CRISPR-associated protein Cas2
3	K15342	cas1	CRISP-associated protein Cas1
4	K07464	cas4	CRISPR-associated exonuclease Cas4 [EC:3.1.12.1]
5	K19091	cas6	CRISPR-associated endoribonuclease Cas6 [EC:3.1.-.-]
6	K01338	lon	ATP-dependent Lon protease [EC:3.4.21.53]
7	K07012	cas3	CRISPR-associated endonuclease/helicase Cas3 [EC:3.1.-.- 3.6.4.-]
8	K19090	cas5t	CRISPR-associated protein Cas5t
9	K19088	cst1, cas8a	CRISPR-associated protein Cst1
10	K19075	cst2, cas7	CRISPR-associated protein Cst2
11	K04096	smf	DNA processing protein
12	K19171	dndD	DNA sulfur modification protein DndD
13	K00399	mcrA	methyl-coenzyme M reductase alpha subunit [EC:2.8.4.1]
14	K00401	mcrB	methyl-coenzyme M reductase beta subunit [EC:2.8.4.1]
15	K00402	mcrG	methyl-coenzyme M reductase gamma subunit [EC:2.8.4.1]
16	K03422	mcrD	methyl-coenzyme M reductase subunit D
17	K02017	modC	molybdate transport system ATP-binding protein [EC:7.3.2.5]
18	K02018	modB	molybdate transport system permease protein
19	K02020	modA	molybdate transport system substrate-binding protein
20	K03057	tfs	transcription factor S
21	K00012	UGDH, ugd	UDPglucose 6-dehydrogenase [EC:1.1.1.22]
22	K06944	K06944	uncharacterized protein
23	K07133	K07133	uncharacterized protein
24	K07133	K07133	uncharacterized protein
**Proteins unique to WWM1085 vs TLL-48-HuF1 (Proteins in blue are also not found in TLL-48 (MAG) and DSM 861^T genomes)**			
1	K09951	cas2	CRISPR-associated protein Cas2
2	K15342	cas1	CRISP-associated protein Cas1
3	K07464	cas4	CRISPR-associated exonuclease Cas4 [EC:3.1.12.1]
4	K19091	cas6	CRISPR-associated endoribonuclease Cas6 [EC:3.1.-.-]
5	K07133	K07133	uncharacterized protein
6	K06921	K06921	uncharacterized protein
7	K06921	K06921	uncharacterized protein
8	K15342	cas1	CRISP-associated protein Cas1
9	K07012	cas3	CRISPR-associated endonuclease/helicase Cas3 [EC:3.1.-.- 3.6.4.-]
10	K09951	cas2	CRISPR-associated protein Cas2
11	K19116	cas5h	CRISPR-associated protein Cas5h
12	K19114	csh1	CRISPR-associated protein Csh1
13	K19115	csh2	CRISPR-associated protein Csh2
14	K07016	csm1, cas10	CRISPR-associated protein Csm1
15	K19138	csm2	CRISPR-associated protein Csm2
16	K09002	csm3	CRISPR-associated protein Csm3
17	K19139	csm4	CRISPR-associated protein Csm4
18	K19140	csm5	CRISPR-associated protein Csm5
19	K19173	dptF	DNA phosphorothioation-dependent restriction protein DptF
20	K19174	dptG	DNA phosphorothioation-dependent restriction protein DptG
21	K19175	dptH	DNA phosphorothioation-dependent restriction protein DptH
22	K17686	copA, ctpA, ATP7	P-type Cu+ transporter [EC:7.2.2.8]
23	K03427	hsdM	type I restriction enzyme M protein [EC:2.1.1.72]
24	K01153	hsdR	type I restriction enzyme, R subunit [EC:3.1.21.3]
25	K07133	K07133	uncharacterized protein
26	K02107	ATPVG, ahaH, atpH	V/A-type H+/Na+-transporting ATPase subunit G/H

Table S8. Physiological characteristics of strain TLL-48-HuF1 and Methanobrevibacter smithii type strains and other human gut strains

		TLL-48-HuF1	*Methanobrevibacter smithii*
Short rod length		1.14 ± 0.4 μm (SD., n=100 cells)	0.99 ± 0.28 μm (SD, n=100 cells)[a]
		Mean OD_{600} (± SD.,n=triplicate cultres)	(+) Growth or (-) no growth reported in Millet et al (1982)
H_2:CO_2 (80:20)	Formate	0.617 ± 0.045*[b]	+
	Acetate	0.837 ± 0.023*[b]	+
	Methanol	0.458 ± 0.021*[c]	0.37 ± 0.021*[ac]
	Trimethylamine	0.254 ± 0.022*[c]	0.315 ± 0.035*[ac]
N_2:CO_2 (80:20)/ Ar:CO2 (50:50)^	Formate	0.063 ± 0.019[b]	-
	Acetate	0.007 ± 0.009[b]	-
	Methanol	0.042 ± 0.008[c]	0.003 ± 0.001[ac]
	Trimethylamine	0.024 ± 0.006[c]	0.032 ± 0.003[ac]

[a] *Methanobrevibacter smithii* DSM 861[T] performed by this study

[b] 3 days incubation

[c] 5 days incubation

*methane concentration was significantly higher ($p<0.05$; unpaired t-test) than the respective N2:CO2 cultures

^Gas mixture used in Miller et al (1982)

Miller, T. L., Wolin, M. J., de Macario, E. C., & Macario, A. (1982). Isolation of *Methanobrevibacter smithii* from human feces. Applied and environmental microbiology, 43(1), 227-232.

Table S9. Primers sequences, amplicon size and annealing temperature used in the study.

Target gene	Primer name*	Sequence (5’-3’)	Ta (°C)	Amplicon size (bp)	Reference
Bacterial 16S rRNA gene	27F	AGA GTT TGA TCM TGG CTC AG	55	~1400	Lane (1991)
	1492R	GGY TAC CTT GTT ACG ACT T			Loy et al (2005)
Methanogen 16S rRNA	Ar84F	GCT CAG TAA CAC GTG GAT AA	58	~1300	This study
	Ar915F	AGG AAT TGG CGG GGG AGC AC	59	~471	Watanabe et al. (2004)
	Ar1386R	GCG GTG TGT GCA AGG AGC	-	-	Skillman et al. (2004)
mcrA	TLL48-mcrA-870F	TCA AGG TTC CAG AGT TTA CC	55	380	This study
	TLL48-mcrA-1250R	GCG AAT GCA GTA GAT GC			
mcrA	mcrA-85F	GGC GGT TGG AAA CAA TCT G	59	1564	This study
	mcrA-1649R	GCT GGG GTG ATG ACA GTT C			

Lane, D.J. 1991. 16S/23S rRNA Sequencing. In: Stackebrandt, E. and Goodfellow, M., Eds., Nucleic Acid Techniques in Bacterial Systematic, John Wiley and Sons, New York, 115-175.
Loy A, Schulz C, Lucker S, Schöpfer-Wendels A, Stoecker K, Baranyi C, Lehner A, Wagner M. 2005. 16S rRNA gene-based oligonucleotide microarray for environmental monitoring of the betaproteobacterial order “Rhodocyclales”. Appl Environ Microbiol 71(3):1373-86.
Watanabe T, Asakawa S, Nakamura A, Nagaoka K, Kimura M. 2004. DGGE method for analyzing 16S rDNA of methanogenic archaeal community in paddy field soil. FEMS Microbiol Lett 232: 153–163.
Skillman LC, Evans PN, Naylor GE, Morvan B, Jarvis GN, et al. 2004. 16S ribosomal DNA-directed PCR primers for ruminal methanogens and identification of methanogens colonising young lambs. Anaerobe 10: 277–285.

Table S10. Hybrid sequencing statistics for strain TLL-48-HuF1

MinIon reads generated	51.38K
N50	11.1kb
Estimated bases	406.92 Mb
sequencing depth (TLL-48-HuF1 genome size 1.95 Mb)	208 ×
Illumina short reads	8,160,838
Effective	99.93%
Sequencing depth (TLL-48-HuF1 genome size 1.95 Mb)	627 x
Unicycler: No. of contigs	1
